# Resuscitation Promotion Factor: A Pronounced Bacterial Cytokine in Propelling Bacterial Resuscitation

**DOI:** 10.3390/microorganisms12081528

**Published:** 2024-07-25

**Authors:** Xinxin Li, Qing Ren, Zhanbin Sun, Yanan Wu, Hanxu Pan

**Affiliations:** School of Light Industry Science and Engineering, Beijing Technology and Business University, Beijing 100048, China; lxx199999@126.com (X.L.); renqing@th.btbu.edu.cn (Q.R.); twins5616@126.com (Z.S.); wyn2769198642@163.com (Y.W.)

**Keywords:** Rpf, resuscitation, growth promotion, peptidoglycan hydrolase, viable but nonculturable state

## Abstract

While confronted with unfavorable growth conditions, bacteria may transform into the dormant state, such as viable but nonculturable (VBNC) state, which is a reversible state characterized by low metabolic activity and lack of division. These dormant cells can be reactivated through the influence of the resuscitation promoting factor (Rpf) family, which are classified as autocrine growth factors and possess peptidoglycan hydrolase activities. To date, with the significant resuscitation or growth promotion ability of Rpf, it has been extensively applied to increasing bacterial diversity and isolating functional microbial species. This review provides a comprehensive analysis of the distribution, mode of action, and functional mechanisms of Rpf proteins in various bacterial species. The aim is to create opportunities for decoding microbial communities and extracting microbial resources from real samples across different research fields.

## 1. Introduction

Bacteria are masters of survival under unfavorable growth conditions. Many bacteria have developed the capacity to enter a non-replicative state with diminished metabolic activities, enabling them to endure a wide range of adverse environments. Such non-replicating states encompass persisters and viable but not culturable (VBNC) bacteria, as well as dormant spores [1,2]. Despite the diverse forms adopted by these non-replicating cells, they often possess a thicker (e.g., increased cross-linking, shortened glycan strands of peptidoglycan) cell wall in order to enhance their robustness to withstand external stress [3,4,5,6]. In the context of the non-replicative state, we employ the term “dormancy” to denote a reversible condition characterized by reduced metabolic activity, allowing a cell to endure prolonged periods without undergoing division [1,7,8]. It has been proposed that dormant cells have the potential to be reactivated into typical, colony-forming bacteria through a specific resuscitation procedure [3,9,10].

Peptidoglycan is present in almost all bacteria cell walls, providing shape, rigidity, and osmotic stability, which serves as a robust protective barrier for bacteria [11]. All peptidoglycans produced by bacteria share the same basic core structure: glycan backbones consisting of alternating N-acetylmuramic acid and N-acetylglucosamine residues, as well as short peptides extending from the lactyl groups of the N-acetylmuramic acid residues [12]. The composition of peptide stems and cross-links exhibits variation across different bacterial species. In Gram-negative species, such as *Escherichia coli*, the peptide sequence is L-Ala-D-Glu-*m*-Dap-D-Ala. Peptides most often connect from the carboxyl group of D-Ala at position 4 of one peptide to the amino group of the *m*-Dap residue at position 3 of another peptide (3–4 cross-link). In Gram-positive species, such as *Micrococcus luteus*, the peptide stem is L-Ala-D-Glu-L-Lys-D-Ala, which connects with another one through a 3–4 cross-link with a bridge consisting of a peptide stem [13]. Specifically, in some special Gram-positive bacteria, such as mycobacteria, the glycan backbone comprises N-acetylglucosamine and both N-acetylmuramic acid and N-glycolylmuramic acid moieties, and the stem peptide consists of D-iGln-*m*-DAP-D-Ala-L-Ala residues with modifications. The cross-link for mycobacterial is a 3–3 cross-link [13]. These peptide bridges cross-link parallel strands to provide a mesh-like structure, for maintaining the integrity of the cell membrane [13]. In dormant cells, peptidoglycan exhibits relatively low reactivity since bacteria stop dividing, while in actively growing cells it displays high turnover [14]. Many dormant cells necessitate the degradation of their robust protective cell wall in order to reinitiate active growth, and various bacteria have developed distinct strategies to accomplish this process. Among them, the so-called “resuscitation promoting factor” (Rpf) has garnered extensive attention.

The Rpf protein was initially identified in *M. luteus* as a bacterial cytokine that facilitates the revival and proliferation of quiescent or dormant cells [15,16]. The resuscitation-promoting ability of Rpf is profound. It was reported that the presence of Rpf protein at a picomolar concentration could increase the number of viable dormant *M. luteus* cells by at least 100-fold [16]. As a muralytic enzyme, similar proteins are widely distributed among other high G+C Gram-positive bacteria, including corynebacteria, mycobacteria, streptomycetes, and some fermicutes (which contain Rpf analogues) [17]. Up to now, Rpfs (largely derived from *M. luteus*) have been extensively employed to promote bacterial growth or resuscitation (not only for *M. luteus* itself, but also for other species), primarily for enhancing microbial diversity and isolating hard-to-culture species [18,19,20], although their functional mechanisms remain incompletely elucidated.

In this review, different aspects of the Rpf proteins including their basic characteristics, applications, structural properties, enzymatic activities, and functional mechanisms have been comprehensively reviewed, aiming to provide updated and in-depth references for researchers and establish a theoretical foundation for decoding the “dark matter” (the dormant majority) of microbial communities in the real world.

## 2. Discovery, Diversity, and Application of Rpf

In natural environments, a large proportion of bacteria are in the dormancy state defined as “a reversible state with low metabolic activity, allowing cells to persist for prolonged periods without undergoing division” [2,21]. For nonsporulating bacteria, it may be considered dormancy regarding the VBNC state [7,9,22]. For such instances, a common feature is their inability to generate colonies on their routine culture media, but dormant cells can be converted to normal, colony-forming bacteria following a special procedure of resuscitation involving cultivation in liquid media, which is termed as “resuscitation” [23,24,25]. Initially, in 1985, Colwell et al. first documented the phenomenon that the growing *M. luteus* entered a quiescent state followed by reactivation of growth [26]. Subsequently, in 1994, Votyakova et al. found that resuscitation and cell growth of starved *M. luteus* cells with lost culturability were favored by the use of the mixture of fresh lactate medium and supernatant from late-logarithmic-phase *M. luteus* cultures as the resuscitation medium, thus suggesting that this recovery is due to the excretion of some factor(s) that promoted the transition of cells from a state in which they are incapable of growth and division to the one of normal state [27]. Later, Mukamolva et al. discovered that *M. luteus* possessed the ability to secrete a factor capable of resuming its growth during its cultivation in the logarithmic growth phase [15]. The protein was then successfully isolated and designated as Rpf [16]. In picomolar concentrations, this protein significantly enhances the viable cell count of dormant *M. luteus* cultures by at least 100-fold and also promotes the growth of viable cells, as well as other high G+C Gram-positive organisms within *Mycobacterium* sp. [16]. Hence, the identification of Rpf and elucidation of its activity signifies a significant milestone in the revival culture of dormant bacteria.

Rpf enzymes were originally defined on the basis of their resuscitation-promoting activities in *Micrococcus* and, later, in *Mycobacterium*. The remarkable attention garnered by Rpfs of *Mycobacterium tuberculosis* stems from their significant implications in both the development and treatment of tuberculosis [28,29]. In subsequent research, Mukamolva et al. identified the widespread presence of the gene responsible for Rpf protein production in other high G+C Gram-positive actinomycetes including *Corynebacterium* sp., *Streptomyces* sp., *Rhodococcus* sp., *Nocardiopsis* sp., *Curtobacterium* sp., and so on [17,30], thus revealing the existence of the Rpf protein family [16,31]. In addition, other Rpf analogues in certain Gram-negative/-positive species have also been reported to exhibit resuscitation-promoting capabilities. For example, the YeaZ protein in *Vibrio parahaemolyticus*, *V. harveyi*, *Salmonella typhimurium*, and *Escherichia coli* has been shown to have promoting effects on bacterial VBNC-state recovery [32,33,34,35]; Lmo0186 and Lmo2522 proteins in *Listeria monocytogenes* are functional equivalents of Rpf, both of which display muralytic activity against crude cell-wall preparations and can reduce lag phase [36]; the recombinant Rpf-like protein from *Achromobacter* sp. HR2 exhibited muralytic activity and demonstrated significant efficacy in resuscitating the growth of VBNC cells, as well as stimulating the growth of normal cells [37]. Overall, the majority of studies have primarily focused on investigating the resuscitation or growth promotion potential of *M. luteus* Rpf, owing to its well-established clarity and effectiveness of action over the past few decades [38,39,40]. In recent years, there has been a noticeable emergence of Rpfs from novel species, exhibiting significant effects on their activity and growth/resuscitation ability [10,41]. By employing such methods, the sources of Rpf can be expanded, but further investigation is needed, through which additional characteristics can be elucidated, thereby enhancing their practical applicability.

With regard to applications of Rpf, extensive research has been conducted on the bacterial resuscitation/growth-promotion ability of Rpfs from different species, as well as their functional mechanisms. The promotion effects at the initial stage were specifically targeted toward individual bacteria [28,38,42,43,44]. To date, there has been a focus on the application of Rpf to enhance bacterial diversity and obtain more functional species from various real environmental samples. To elucidate the bacterial community structure, sequence-based techniques such as 16S rRNA sequencing and metagenomic analysis were commonly employed, referred to as non-culture-based methods [20]. Occasionally, these methods were complemented with culturomics analysis to comprehensively illustrate the promotion effects of Rpf. On the one hand, specific bacterial genera were enriched upon Rpf treatment, many of which were associated with bioremediation in polluted environmental samples [18,19,45,46]. On the other hand, the resuscitation ability of Rpf led to the isolation of species with specific functions or novel species. For example, 51 potentially novel bacterial species were isolated from soil [47]; 2 rare actinobacteria were also obtained from soil after Rpf treatment [48]; Shi et al. isolated *Achromobacter* sp. HR2 that exhibited effective degradation of Aroclor 1242 [49]. Collectively, the application of Rpfs and their positive effects are presented in Table 1.

## 3. Structural Characteristics of Rpf Protein Family

**Figure 1:**
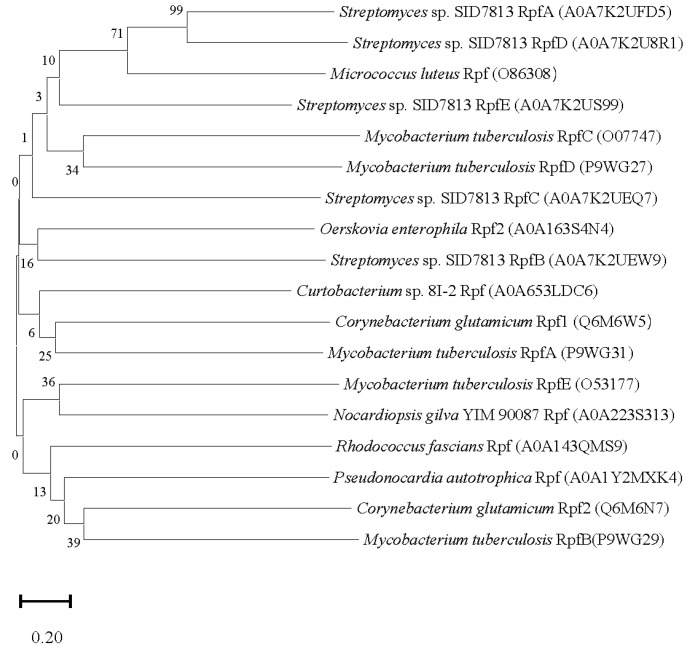
Phylogenetic analysis of Rpfs from different bacteria. The phylogenetic tree was conducted based on amino acid sequences of Rpfs via Mega 11 software using the neighbor-joining method. Bootstrap values were based on 1000 replications. Bar, 0.2 means substitutions per nucleotide position.

Do the Rpfs, with their diversity, exhibit similar functionality? Given the close correlation between protein function and structure, a phylogenetic analysis was firstly performed on Rpfs from major bacterial genera (Figure 1), based on their primary structural characteristics. The protein sequences of Rpfs exhibit significant divergence across different genera (Figure 1). Among these, RpfA and RpfD from *Streptomyces* sp. exhibit the highest degree of similarity, forming a distinct clade with *M. luteus*, implying that these proteins demonstrate relatively greater sequence homology (Figure 1). Additionally, it is a prevalent occurrence for bacteria to contain multiple Rpf proteins, as observed in species such as *Corynebacterium glutamicum*, *M. tuberculosis*, and *Streptomyces* sp. The protein sequences of Rpfs from a specific strain can exhibit significant variation, as seen in the case of Rpf1 and Rpf2 in *C. glutamicum*, Rpf B/C/E in *Streptomyces* sp. SID7813, and RpfA-E in *M. tuberculosis (*Figure 1). This suggests that different Rpfs within a specific microorganism may possess distinct spatial structures, thereby potentially playing diverse roles in promoting bacterial resuscitation/growth.

While the primary structures of Rpf from different species exhibit significant variation, their active domains share some common characteristics. The majority of Rpfs possess a conserved domain with specific functional attributes. Structural prediction of this domain suggests that it primarily consists of a lysozyme-like domain (c-type lysozyme fold), LysM domain, and G5 domain (Table 2). The presence of a lysozyme-like domain suggests that Rpfs may have lysozyme-like activity or even bind saccharides [62]. The LysM modules consist of 43–50 amino acids that adopt a highly conserved βααβ-fold, with particularly high sequence conservation in the first 16 residues [63]. Prokaryotic LysM modules bind peptidoglycan, the main component of the bacterial cell wall [64]. Similarly, the G5 domain may confer localization or substrate specificity on the proteins, which is typically present in proteins involved in bacterial cell-wall metabolism [65]. Taking these factors into consideration, it is possible that Rpf could potentially stimulate bacterial resuscitation or growth by facilitating the hydrolysis of cell-wall peptidoglycan.

Experimental investigations into the active domains of Rpfs have provided additional validation for the predicted outcomes. Cohen-Gonsaud et al. reported on the solution structure of the Rpf domain from *M. tuberculosis* Rv1009 (RpfB) solved by heteronuclear multidimensional Nuclear Magnetic Resonance (NMR) [66]. Results revealed that the Rpf domain is a compact hybrid of the soluble lytic transglycosidase and c-type lysozyme folds, both of which cleave peptidoglycan. A few crucial features such as the catalytic glutamate are conserved, which is critical for resuscitation activity [66]. It has also been found in a study on the structure of the conservative domain RpfB from *M. tuberculosis* by NMR that the conservative domain has a spatial organization similar to the tertiary structure of lysozyme C [67,68]. The conservative domain of Rpf is characterized by six α-helices and one β-sheet, which are spatially arranged in a manner identical to the α-helical regions of lysozyme C and certain lytic enzymes involved in bacterial cell-wall metabolism [67,68]. Subsequently, X-ray crystallographic analysis revealed that the catalytic domain of RpfB retains all essential catalytic residues for lysozyme activity and exhibits six peptidoglycan-binding sites, akin to various types of lysozymes. As a result, the catalytic domain was characterized as a “mini-lysozyme” [69]. Additionally, the 3D structural information of Rpfs is also presented in Table 2, which displays high diversity. Hence, structural studies of Rpf unequivocally demonstrate their classification as peptidoglycan hydrolases, implying that potential enzymatic activity may be crucial for the observed physiological impacts.

## 4. Enzymatic Activity of Rpf

Based on the structural characteristics of Rpfs, their catalytic domains demonstrate their similarity to lysozyme and to lytic transglycosylases—the groups of enzymes that cleave the β-1,4-glycosidic bond between N-acetylmuramic acid and N-acetylglucosamine. In order to elucidate the functional mode of the proteins, experimental assays were performed to assess their enzymatic activities.

As a founding member of the Rpf family, *M. luteus* Rpf was proved to be a muralytic enzyme, as revealed by its ability to cause lysis of *E. coli*, release fluorescent material from fluorescamine-labeled cell walls of *M. luteus* and hydrolyze the artificial lysozyme substrate of 4-methylumbelliferyl-β-D-N,N′,N″-triacetylchitotrioside while expressed and secreted into the periplasm [70]. It has been demonstrated that substitution of the putative catalytic glutamate in Rpf protein from *M. luteus* with glutamine (E54Q) resulted in only a partial loss of activity [38,42,68], while substitution with alanine (E54A) or lysine (E54K) effectively suppressed the enzymatic activity [70]. The activity was significantly inhibited in a mutant Rpf with two substituted cysteine residues that could potentially contribute to the formation of a functionally crucial intramolecular disulfide bond, but the substitution of either Cys53 or Cys114 separately resulted in only a partial decrease in activity [66,70].

In *Rhodococcus* sp., the RpfB protein of *Rhodococcus* sp. (GX12401) had the highest homology with *M. tuberculosis* RpfB [40]. When 4-methylumbelliferyl-β-D-N,N′,N″-triacetylchitoside was employed as the enzyme substrate for assessing lysozyme activity, it was observed that the recombinant protein RpfB exhibited robust stability and enzymatic efficacy [40]. In the case of *R. erythropolis* KB1, Rpf-1 exhibited muralytic and weak protease activities, with the former being responsible for its growth promotion and resuscitation effects [71]. Moreover, substitution of the conserved amino acid residue Gln69 resulted in a significant reduction in muralytic activity, highlighting the pivotal role of Gln69 in enzyme function [71].

For *Streptomyces coelicolor*, Rpfs (A–E) were found to promote spore germination and are critical for growth under nutrient-limiting conditions, and in vitro biochemical assays revealed various levels of peptidoglycan cleavage capabilities for each Rpf [72]. The two domains of LysM and LytM, typically existing in lysozyme and lytic transglycosylase, respectively, share the same substrate specificity [73]. However, lysozyme catalyzes a hydrolysis reaction that requires water to break their cognate glycosidic bonds, and lytic transglycosylase cleaves peptidoglycan with the concomitant formation of an intramolecular 1,6-anhydromuramoyl product [13,73]. Sexton et al. (2020) conducted an assessment of the activity of all five Rpfs from *S. coelicolor* (RpfA–E) by hydrolyzing *M. luteus* peptidoglycan suspended in 18O-labeled water to specify their activities. Upon identification of the products, the results indicated that RpfA/B/C/E each functioned as lytic transglycosylases rather than muramidases or β-N-acetylglucosaminidases [74]. Additionally, a mutagenesis assay revealed that their LysM and LytM domains enhanced the enzyme activity of Rpf [74].

## 5. Functional Mechanism of Rpf

### 5.1. Rpf Functions with Its Partner RipA

**Figure 2:**
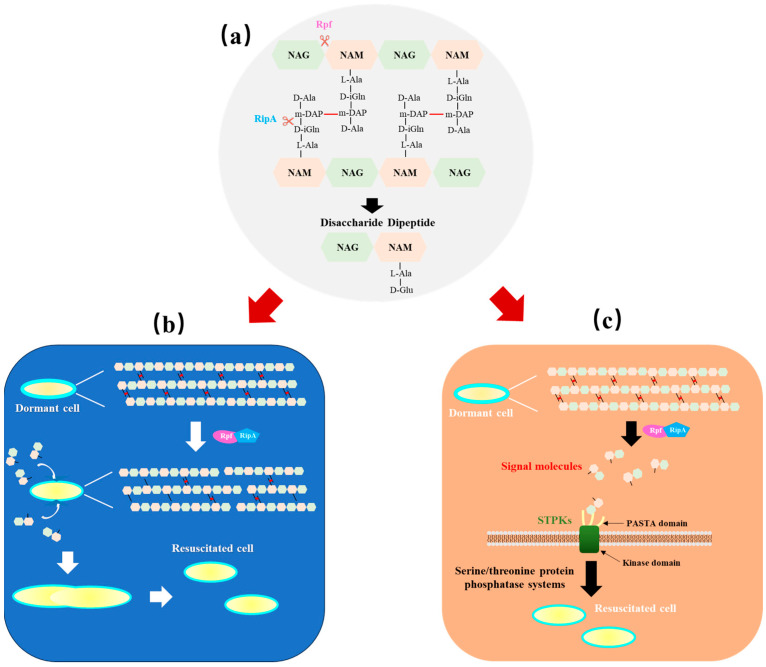
Proposed role of Rpf in remodeling of peptidoglycan and production of muropeptide signaling molecules to promote resuscitation. (**a**) Rpf functions with its partner RipA. Rpf is predicted to cleave the β-1,4 glycosidic bond between NAM and NAG in the glycan backbone, in a manner similar to lytic transglycosidases or lysozymes, whereas RipA cleaves bonds in the peptide stems. (**b**) Remodeling of bacterial cell wall to stimulate growth or resuscitation by Rpf. Rpf and RipA work synergistically to break down the thick protective cell wall for revival, which is followed by accommodation of the insertion of new peptidoglycan units. (**c**) Signaling molecules of peptidoglycan fragments released by Rpf-stimulating resuscitation. Muropeptide signaling molecules, the Rpf and RipA hydrolysis product, can bind to the extracellular sensor domain of STPKs including PrkC, PknB and then be phosphorylated to trigger resuscitation. All the arrows (black, red and white) represent the direction of the flow.

The structural characteristics and biochemical activities described above suggest that the enzymatic hydrolysis of peptidoglycan, facilitated by Rpf, may play a crucial role in promoting bacterial growth and resuscitation. Numerous enzymes are involved in the synthesis and degradation of peptidoglycan, working in conjunction with other enzymes [75]. Through screening in a yeast two-hybrid system, Hett et al. discovered that the function of RpfB from *M. tuberculosis* is associated with an endopeptidase RipA (resuscitation-promoting factor-interacting protein) [76]. RipA is a secreted, cell-associated protein, which localizes to the septa of the bacterial cell and is indeed a peptidoglycan hydrolase that is capable of digesting cell-wall material [76]. Deletion of the gene encoding RipA was also demonstrated which resulted in a reduction in growth rate and the development of anomalous morphology, such as branching and formation of chains, in *M. tuberculosis* and *M. smegmatis* cells [77]. These findings suggest that RipA is likely to play a crucial role during the final stage of cell division [78]. It is common for certain bacterial species to contain multiple Rpf proteins; however, not all of these Rpfs have been thoroughly investigated. Therefore, the subsequent sections will primarily focus on the already studied Rpfs. In terms of enzymatic activities of the two proteins, RpfB is known to cleave the glycoside bonds of the cell-wall peptidoglycan [66], while RipA is predicted to function as an endopeptidase that cleaves the stem peptide [D-iGlu-meso-diaminopimelic acid (Dap)] [79,80]. The interaction between RipA and RpfB at the septum of dividing cells has been shown to positively regulate peptidoglycan hydrolysis, as evidenced by the synergistic degradation of PG in vitro by the RipA–RpfB complex (Figure 2a) [76,79]. RpfE can also interact with RipA [75].

### 5.2. Rpf May Participate in the Remodeling of the Bacterial Cell Wall to Stimulate Growth or Resuscitation

In harsh environments, bacteria often modify their cell-wall structure as a means of adaptation. For example, actinomycetes tend to produce dormant exospores with thick cell walls to withstand adverse factors, resulting in difficulty germinating and growing under laboratory conditions [70,79]; VBNC-state bacteria may encounter a series of peptidoglycan modifications such as high degree of cross-linking, an increase in DAP–DAP cross-linking, and a shortening of the average length of glycan strands, in comparison with dividing cells, resulting in a more robust cell-wall structure [5,6,81]. In such conditions, a return to active growth requires the breakdown of the thick protective cell wall facilitated by Rpf proteins. The mechanism of action of Rpf remains a topic of debate. The preceding sections have indicated that the Rpf and RipA complex has the potential to function during peptidoglycan hydrolysis. Consequently, some researchers thought that the growth promotion or resuscitation stimulation capability of Rpf may be attributed to the remodeling of the bacterial cell wall. It was emphasized that Rpf activity in *S. coelicolor* was not correlated with peptidoglycan-responsive Ser/Thr kinases for cell signaling, which thus suggested that in Streptomyces, Rpfs had a structural rather than signaling function during spore germination [72,74]. Some Rpfs exhibit lytic transglycosylase activity, which facilitates the cleavage of the glycan backbone to accommodate the insertion of new peptidoglycan units during growth and is implicated in cell-wall remodeling processes such as envelope spanning (Figure 2b) [82,83].

As proteins related to the hydrolysis of cell-wall peptidoglycan, the remodeling of peptidoglycan by Rpfs must be rigorously regulated in order to maintain the structural integrity of the cell wall. In *M. tuberculosis*, the activity of RpfB–RipA is negatively impacted by its interactions with penicillin binding protein 1 (PBP1) [76]. The promotion of germination and growth of Streptomyces coelicolor is attributed to a unique mode of enzymatic autoregulation, which is mediated by a domain (DUF348) located within the N-terminus of the protein. Removal of this domain resulted in significantly enhanced peptidoglycan cleavage capacity. Therefore, it is speculated that the association between RipA and RpfB may induce a conformational change in RpfB, thereby alleviating the DUF348-mediated inhibition of enzyme activity [72].

Collectively, Rpf may function as a hydrolase, leading to restricted hydrolysis of the modified peptidoglycan in dormant cells. This process may promote cell-wall synthesis and growth, finally stimulating the initiation of the division process for resuscitation.

### 5.3. Rpf-Released Peptidoglycan Fragments Function as Signaling Molecules to Stimulate Growth/Resuscitation

The structure of a mycobacterial peptidoglycan synergistic hydrolysis product under the influence of RpfB-RipA comlex was identified to be N-acetylglucosaminyl-β(1→4)-N-glycolyl-1,6-anhydromuramoyl-L-alanyl-D-isoglutamate (anhydroMurNAc) [75]. It was further revealed that the disaccharide-dipeptide fragments represent the minimal structural unit capable of reviving dormant mycobacterial cells [75,84].

Whether Rpf activity promotes resuscitation by simply remodeling the dormant cell wall or by generating peptidoglycan fragments (muropeptides) that function as signaling molecules has been the subject of much debate. Regarding the stimulation mechanism of the peptidoglycan fragments, some researchers argue that bacteria can be revived from a non-growing state through the sensing of specific peptidoglycan fragments by signaling pathways [84]. For example, bacteria always apply eukaryotic-like serine/threonine protein kinases (STPKs) and associated serine/threonine protein phosphatase systems to bind and respond to peptidoglycan [85]. STPKs are monomeric transmembrane polypeptides with an extracellular sensor domain and an intracellular cytoplasmic protein kinase domain, with which phosphorylates target proteins upon recognition of a specific signal, leading to a coordinated cellular response [83]. The typical serine/threonine protein kinase found in *Bacillus subtilis*, PrkC, which has an external domain consisting of three repeat PASTA (penicillin-binding protein and serine/threonine kinase-associated) domain motifs, has been implicated in the regulation of growth through its modulation of sporulation and biofilm formation [86]. During active growth, it was shown that disaccharide-dipeptide fragments serve as potent germinants for *B. subtilis* spores, requiring the involvement of PrkC in peptidoglycan binding and the initiation of a signal cascade leading to spore germination [87]. 

In mycobacteria, PknB is the homologue of PrkC, which is the essential STPK [88]. PknB shares a high degree of structural similarity to other STPKs, with an intracellular kinase domain as well as an extracellular sensing domain containing four PASTA motifs, suggesting that this protein is also able to bind peptidoglycan [89]. Therefore, in conjunction with the biochemical role of Rpfs in the modification of cell-wall structure, it is conceivable that Rpfs and other enzymes synergistically function to generate disaccharide-dipeptide fragments as signaling molecules, which can bind to PknB or other PrkC homologues to trigger resuscitation in a manner akin to spore germination in *B. subtilis* [87]. However, several components of this mechanism are currently based on hypotheses, and further investigation is required to obtain more direct evidence in future studies.

## 6. Prospections

Here in this review, the diversity, application, structural characteristics, and functional mechanisms of Rpfs are comprehensively reviewed. Rpfs have garnered significant interest for their ability to enhance bacterial diversity and isolate “hidden” bacterial species, due to their functional feature of exerting a substantial effect at low concentrations. To date, the focus of Rpf treatment has primarily been on environmental samples. Broadening the research scope to include additional areas such as the food industry and agriculture may potentially open up a novel avenue, although it is essential to thoroughly assess any associated risks. Moreover, another important task for the future is the elucidation of the nature and action preference of the Rpfs, as well as the mechanisms of their action.

## Figures and Tables

**Table 1:** The utilization of Rpf proteins from diverse sources in bacterial resuscitation.

Bacterial Origin of Rpf	Experimental Sample	Promotive Effects
*M. luteus*	*Mycobacterium tuberculosis*	Rpf stimulates *M. tuberculosis* viability measured by the MPN method [35].
	*M. luteus*	Pronounced influence on the true lag phase and cell growth on lactate minimal medium [50].
	Polychlorinated biphenyls-contaminated soils	The enrichment culture produced by the addition of SRpf significantly enhanced biphenyl degradation efficiency, cell growth, and bacterial diversity [51].
	Activated sludge	The phyla of Proteobacteria and Actinobacteria, which are closely related to biological nutrient removal, were greatly abundant after SRpf addition [45].
	Activated sludge	Resuscitated functional bacterial community for enhancing biodegradation of phenol linked mainly to the genus *Corynebacterium* and the genera *Proteiniphilum* and *Petrimonas* [46].
	Activated sludge	Two strains from genera *Bacillus* and *Corynebacterium* could enhance phenol degradation under high salinity conditions [52].
	Sediments from a nitrogen-polluted river	From URS and LRS, a total of thirteen strains with heterotrophic nitrification ability were resuscitated by Rpf addition, which belonged to genera *Bacillus*, *Pseudomonas*, *Stenotrophomonas*, and *Acinetobacter* [53].
	*Helicobacter pylori*	Showed high resuscitation effect on kanamycin-induced *H. pylori* coccoid forms [36].
	MSM medium with reactive blue 19	Immobilized *Bacillus* sp. JF4 isolated by Rpf could effectively decolorize anthraquinone dye RB 19 [18].
	Liquid culture with aroclor 1242	Exogenous Rpf stimulated endogenous *rpf* expression in *Rhodococcus biphenylivorans* TG9^T^, leading to the resuscitation of VBNC TG9^T^ cells, which finally contributed to its polychlorinated biphenyls-degrading capacity [54].
	Soils polluted with used lubricant oils (ULOs)	Bioremediation of ULOs was significantly enhanced, which was attributable both to the reactivation of hydrocarbonoclastic bacterial genera (e.g., *Pseudomonas*, *Comamonas*, *Stenotrophomonas*, and *Gordonia*) [19].
	Soil containing field mustard	rRpf reduced soil respiration, decreased bacterial abundance, and increased fungal abundance [55].
	Soil	A total of 51 potentially novel bacterial species were isolated after SRpf treatment [47].
	Polychlorinated biphenyl (PCB)-contaminated soils	The rRpf-responsive population was mostly represented by *Sphingomonas* and *Pseudomonas*, which are most likely the key PCB degraders [56].
	Soil	Two rare actinobacteria were obtained in the experimental group supplemented with rRpf protein [48].
	Air	Increased bacterial culturability by up to 30% when synergistically employed Reasoner’s 2A agar (R2A) and R2A + Rpf media [37].
	Cockroach gut and soil	Promoted the cultivation of a diverse set of bacteria and in particular certain clades of the phyla Actinomycetota and Bacillota [20].
	Anaerobic mineral salt medium with PCBs	Enhanced PCB dechlorination 1.2–14.1 times higher than control cultures, and *Desulfobacterota* and *Bacteroidetes* were Rpf-responsive non-dechlorinators [57].
	PCB-contaminated soils	The dechlorinators, including *Dehalococcoides* in *Chloroflexi* and *Desulfitobacterium* in *Firmicutes*, were greatly enriched via rRpf amendment [58].
*Rhodococcus rhodochrous*	*R. rhodochrous*	MPN count was close to the total cell count by incorporating supernatant taken from growing cultures [42].
	*R. rhodochrous*, *R. fascians*, *M. luteus*	Growth was promoted while incubated with culture supernatant [43].
*Rhodococcus* sp. (GX12401)	*Rhodococcus* VBNC cells	*Rhodococcus* VBNC cells were resuscitated with 1 pM rRpf treatment, which increased by 18% compared with the control [40].
*R. fascians*	*R. rhodochrous*, *R. fascians*, *M. luteus*	Growth was promoted while incubated with culture supernatant [43].
*Mycobacterium tuberculosis*	*M. bovis*	Actively growing cells of M. bovis (BCG) did not respond to these proteins, whereas bacteria exposed to a prolonged stationary phase did [38].
	*M. tuberculosis*	MPN count value was further increased by one log using supernatant from an actively growing culture [42].
		RpfD could efficiently stimulate the resuscitation of *M. tuberculosis* H37Ra, and the rabbit anti-RpfD serum could completely inhibit this effect [59].
*Corynebacterium glutamicum*	*C. glutamicum*	The growth of *C. glutamicum* was impaired in Δ*rpf1* and Δ*rpf2* strains; a significant shortening of the apparent lag phases was observed after addition of culture supernatants [44].
*Tomitella biformata* AHU 1821T	*T. biformata*	Amounts of 2 and 20 μg/mL recombinant Rpf protein resuscitated non-dividing *T. biformata* cells [60].
	Melted surface sterilized ice wedge samples	Samples treated by 1250 pM rRpf had a 14-fold higher number of CFUs on agar plates after 8 d incubation [61].
*Nocardiopsis halophila*	*Nocardiopsis halophila*	Resuscitation effect on the VBNC *N. halophila* was most evident when 0.1 mg/mL of the rRpf protein was added to the medium [28].
*Listeria monocytogenes*	*lmo0186 lmo2522* double mutant of *L. monocytogenes*	Lmo0186 and Lmo2522 (equivalent to Rpf) stimulated the growth of *lmo0186 lmo2522* double mutant that was growth-arrested [33].

SRpf: culture supernatants from *M. luteus* containing Rpf. rRpf: recombinant Rpf.

**Table 2:** Rpf structural diversity in bacteria.

Species	Protein	Protein Length/AA	Domain	3D Structure
*M. luteus*	Rpf (O86308)	223	172–220: LysM domain	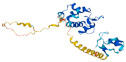
*M. tuberculosis*	RpfA (P9WG31)	407	41–114: Transglycosylase-like domain	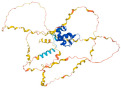
	RpfB (P9WG29)	362	192–272: G5 domain	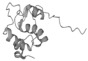
	RpfC (O07747)	176	70–140: Transglycosylase-like domain	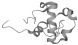
	RpfD (P9WG27)	154	50–121: Transglycosylase-like domain	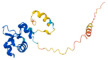
	RpfE (O53177)	172	98–168: Transglycosylase-like domain; 25–84: PRK12438 super family	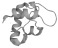
*Corynebacterium glutamicum*	Rpf1 (Q6M6W5)	193	37–111: Resuscitation-promoting factor core lysozyme-like; 117–190: Rpf1 C-terminal domain	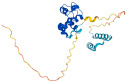
	Rpf2 (Q6M6N7)	374	210–290: G5 domain	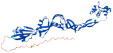
*Streptomyces* sp. SID7813	RpfA (A0A7K2UFD5)	244	45–117: Transglycosylase-like domain 195–243: LysM domain	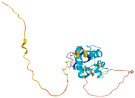
	RpfB (A0A7K2UEW9)	458	183–211: DUF348 domain 291–371: G5 domain	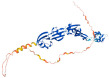
	RpfC (A0A7K2UEQ7)	341	285–334: LysM	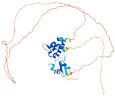
	RpfD (A0A7K2U8R1)	439	165–214: LysM	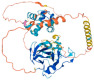
	RpfE (A0A7K2US99)	121	48–121: Transglycosylase-like domain	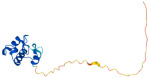
*Rhodococcus fascians*	Rpf (A0A260UFL0)	375	205–285: G5 domain	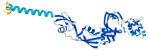
*Curtobacterium* sp. 8I-2	Rpf (A0A653LDC6)	234	184–232: LysM domain	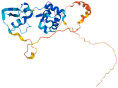
*Pseudonocardia autotrophica*	Rpf (A0A1Y2MXK4)	134	57–127: Rpf core lysozyme-like domain	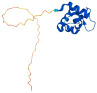
*Oerskovia enterophila*	Rpf2 (A0A163S4N4)	434	239–320: G5 domain	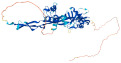
*Nocardiopsis gilva*	Rpf (A0A223S313)	206	126–200: Rpf core lysozyme-like domain	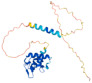

AA: amino acid. LysM: the domain recognizes polysaccharides containing N-acetylglucosamine (GlcNAc) residues including peptidoglycan, an essential component of the bacterial cell wall. G5: the domain may confer localization or substrate specificity on the proteins in which it is found. The numbers listed in the domain column represent amino acid positions of the domains. Information on protein name, length, domain, and 3D structure was found in the Uniprot database (https://www.uniprot.org/, accessed on 31 May 2024). The codes under the protein name are accession numbers of the Rpfs in Uniprot.
